# Opportunities and challenges in conducting secondary analysis of HIV programmes using data from routine health information systems and personal health information

**DOI:** 10.7448/IAS.19.5.20847

**Published:** 2016-07-20

**Authors:** Stephen Gloyd, Bradley H Wagenaar, Godfrey B Woelk, Samuel Kalibala

**Affiliations:** 1 Department of Global Health, University of Washington, Seattle, WA, USA; 2 Health Alliance International, Seattle, WA, USA; 3 Elizabeth Glaser Pediatric AIDS Foundation, Washington, DC, USA; 4 HIVCore/Population Council, Washington, DC, USA

**Keywords:** secondary data analysis, health information systems, personal health information, electronic medical records, data accuracy, missing data, data analysis

## Abstract

**Introduction:**

HIV programme data from routine health information systems (RHIS) and personal health information (PHI) provide ample opportunities for secondary data analysis. However, these data pose unique opportunities and challenges for use in health system monitoring, along with process and impact evaluations.

**Methods:**

Analyses focused on retrospective case reviews of four of the HIV-related studies published in this JIAS supplement. We identify specific opportunities and challenges with respect to the secondary analysis of RHIS and PHI data.

**Results:**

Challenges working with both HIV-related RHIS and PHI included missing, inconsistent and implausible data; rapidly changing indicators; systematic differences in the utilization of services; and patient linkages over time and different data sources. Specific challenges among RHIS data included numerous registries and indicators, inconsistent data entry, gaps in data transmission, duplicate registry of information, numerator-denominator incompatibility and infrequent use of data for decision-making. Challenges specific to PHI included the time burden for busy providers, the culture of lax charting, overflowing archives for paper charts and infrequent chart review.

**Conclusions:**

Many of the challenges that undermine effective use of RHIS and PHI data for analyses are related to the processes and context of collecting the data, excessive data requirements, lack of knowledge of the purpose of data and the limited use of data among those generating the data. Recommendations include simplifying data sources, analysis and reporting; conducting systematic data quality audits; enhancing the use of data for decision-making; promoting routine chart review linked with simple patient tracking systems; and encouraging open access to RHIS and PHI data for increased use.

## Introduction

Massive amounts of data have been collected in low- and middle-income countries for HIV programmes over the past several decades. Complex data collection requirements have come from donors, multilateral organizations and ministries of health to monitor their substantial HIV investments [1,2]. To calculate an array of HIV-related tracking indicators, groups have used data from routine health information systems (RHIS), personal health information (PHI), community sample surveys, demographic surveillance sites and special studies.

In this context, RHIS and PHI data provide ample opportunities for secondary data analysis. RHIS data typically come from monthly reports generated at health facilities derived from service-specific registry books. These data are capable of providing accurate, reliable, timely, representative and continuous information on the health system and patients in the system [3–5]. They can be real-time indicators of service coverage and quality, and the numerous repeated observations over extended periods of time can provide robust data for secondary analysis, including quasi-experimental designs, such as controlled interrupted time-series analysis, which provide strong inferences of causality in measuring the impact of programme and policy changes. These facility-level data can also provide knowledge of geographic variation – highlighted as important for focusing on the UNAIDS agenda to “leave no-one behind” [6], the World Health Organization (WHO) guidelines for the expansion of antiretroviral therapy (ART) [7] and the PEPFAR 3.0 objectives of doing the “right thing, right place right time” [8].

PHI typically includes facility-based patient charts that are either paper-based or electronic medical records (EMR), personal pharmacy pick-up records and/or home-based booklets (e.g. mother-child health booklets). Paper charts remain the mainstay for most patient information; EMR systems are usually managed by international implementing partners and harmonized across countries in which a partner works [9]. EMR data are increasingly used for longitudinal studies that examine enrolment and retention in HIV care [10].

Secondary data represent the low hanging fruit for cost-effective programme evaluation for improving the quality of HIV services. When RHIS and PHI are available and accurate, expensive data collection to assess health system performance can be avoided [11–13]. Furthermore, RHIS and PHI can enable making inferences at the health facility level, in contrast to most intermittent sample surveys, special studies or demographic surveillance sites which most often are not powered to be actionable below the provincial level. RHIS and PHI can be used to guide decision-making at national and sub-national levels, yet problems with accuracy and completeness of the data often undermine their usefulness [14]. Case studies of poor RHIS data are widespread and the use of RHIS is frequently disparaged [15–17]. However, many studies have shown that rapid and relatively low-cost methods of improving data completeness and reliability are highly effective [3,4,18–23].

The aim of this paper is to review the accuracy of RHIS and PHI data collected and used in four studies of the HIVCore project, all published in this JIAS supplement. We believe that these studies, each carried out in a different country, provide a broad spectrum of experiences using RHIS and PHI for secondary data analysis. Our objective is to identify key challenges regarding their use and to identify ways to address those challenges going forward, contextualized in the broader literature [24–28].

## Methods

The studies reviewed in this article were conducted across four countries between 2012 and 2015. All of the countries have a substantive PEPFAR presence that includes large international NGO implementing partners and heavy data reporting requirements – a situation that is common in PEFAR-supported countries in sub-Saharan Africa. Each of the studies was conducted with support of the implementing partners. The authors independently reviewed each of the studies and used consensus decision-making to identify themes to achieve a unifying analysis of the studies. Communication was carried out through iterative phone calls, emails and sharing of manuscript drafts.

1. **Prevention of mother-to-child transmission (PMTCT) cascade assessment in Cote d'Ivoire** [24] identified loss to follow-up and associated factors in the Côte d'Ivoire PMTCT programme, using a nationally representative, cross-sectional sample of 30 randomly selected health facilities providing PMTCT. The quantitative aspect of the study assessed 13 indicators from PMTCT-related registries and patient charts to determine the magnitude of loss to follow-up among 1741 HIV-positive women at multiple steps in the PMTCT cascade and compared high- and low-performing sites to identify factors associated with differences in PMTCT performance.
2. **PMTCT retention assessment in Rwanda** [25] investigated levels of retention along the PMTCT cascade among HIV-positive pregnant women and their infants attending EGPAF-supported health facilities using a retrospective cohort analysis among 474 women in 12 health facilities. Data were linked from ANC, PMTCT, labor and delivery, HIV Exposed Infants, postnatal care, ART and Exposed Infant Diagnosis registers.
3. **Task shifting and ART retention in Uganda – cost analyses** [26,27] analyzed annual ART-related costs among the three large AIDS organizations, each representing a task-shifting model. The study team collected cost information regarding ARV drugs, non-ARV drugs, ART-related lab tests, personnel and administrative costs. Data for the analysis came from patient charts and included the client's date of initiation of ART and ARV refill visits to determine attendance and retention.
4. **Assessment of linkages from HIV testing to enrolment and retention in care in central Mozambique** [28] assessed enrolment and retention of HIV-positive individuals through assessment of HIV registries at 87 health facilities in Central Mozambique, and review of 795 patient charts of HIV patients conducted at eight health facilities offering ART. Quantitative data were abstracted from facility monthly reports, HIV registries and patient charts, and adjusted to account for missing and inconsistent values to measure losses to follow-up. ART registries were linked to patient charts via unique patient codes. No unique patient codes were used for HIV testing.

## Results

The authors identified several general themes during the analysis of the studies, including missing, inconsistent and implausible data; changing indicators; differential utilization of services; and data linkages.

### Incomplete, missing and implausible data

This was by far the most common challenge encountered in all of these studies, both in RHIS and PHI. Incomplete RHIS included missing monthly reports or implausible data in the submitted reports or in the registries on which the monthly reports were based. Incomplete PHI included key information regarding demographics, initiation of treatment, clinic visits, CD4 counts, date of birth and infant information among PMTCT patients. Examples of incomplete, missing and implausible data are described for each study as follows:

The Cote d'Ivoire PMTCT study noted that missing data were widespread in the entire national reported sample. For the 11 registry indicators that were assessed to evaluate the PMTCT cascade, 24 of the 30 study sites had one or more months with no data during the 12-month study period. In addition, PMTCT registries, ART registries and patient charts were incorrectly filled out at 10, 9 and 14 sites, respectively, out of the 30 total sites. The study team found large variability in data completeness from different sites. Moreover, there were major inconsistencies in similar indicators from different sources. For example, the recorded proportion of women tested for HIV was much less in the mother-child vaccination booklets (84%) than in PMTCT testing registries (95%) at the same sites. In addition, at every site, on-site registry indicators were inconsistent with the same indicators in the national database. On average, more than half of all indicators compared had a discrepancy of larger than 5% between on-site data and the same data in the national database. The data quality and burden of having too many indicators in multiple registries was highlighted as a key finding of the study. In spite of the data weaknesses, authors were able to triangulate data from multiple sources to attain usable estimates for the study analysis.

The Rwanda PMTCT study demonstrated similar challenges with missing key data. The type of regimen was unknown for 20% of the women on ARV. For the infants, 38% of infant files were not found. Among the patient charts, the type of regimen was unknown for nearly half of the women who were reported to be on lifelong ART. In the maternity, postnatal care and HIV-exposed infant registries were used to link the mother-baby pairs. Many of the infant records were missing information, including place of delivery (40% missing), mode of delivery (70% missing) and gender (58% missing).

The Uganda study excluded four of 29 health facilities from one of the three samples because of missing and implausible data. Two additional AIDS service organizations were excluded whose data were inadequate for comparison after data cleaning. Study teams noted frequent duplicate entries of data, dates entered in incorrect formats, including visits falling outside the eligible enrolment period and mismatched site codes. Implausible client histories showed that clients received, in the aggregate, over 125% of ART drugs based on their follow-up period. Many errors necessitated the exclusion of records from the final analysis. In two sites, 78% of all collected client histories failed to meet one of four essential criteria for adherence and only 67 of 304 patient histories were sufficiently complete for analysis for the study. As with other studies, missing data were highly variable among indicators: 64% of all unique client histories were missing drug delivery numbers, regimen information or both; and 92% of all unique client histories were missing one or more scheduled appointment dates. Moreover, large variability in missingness was found between sites.

The Mozambique HIV linkages study demonstrated that missing data and data inconsistencies were common in all stages of the treatment cascade, including testing, enrolment and retention in treatment. Over 46% of the 1944 monthly HIV testing reports expected from the health facilities were missing from the national RHIS. Most HIV testing reports submitted did not report from more than several of the 13 clinical programmes where HIV testing might have occurred. Registration in pre-ART care was more reliably reported; less than 5% of these monthly reports were missing. The much lower missingness among numerators (pre-ART registration) than denominators (HIV testing) caused a large overestimate of the likely true proportion of people tested HIV positive who were enrolled in pre-ART. After the authors imputed data from non-missing months to estimate the missing months, the overall estimated proportion of people tested HIV positive registered in pre-ART dropped from 97 to 75%.

Another challenge from the Mozambique HIV linkages study was that registration in pre-ART care did not necessarily imply enrolment in HIV care. Although patient charts were required for all people that tested HIV positive who registered in care, charts were found in only 66 and 60%, respectively, of patients who had been registered in ART and pre-ART – with a large variation among facilities (41–92% of ART charts found). Patient charts and pharmacy records inconsistently recorded individual patient visits. Only 37% of patient charts that were found demonstrated evidence of active retention. Given the losses in all steps of the ART cascade, the total retention of people tested HIV positive identified in the system was only 18% in a sample whose mean time in treatment was 18 months – when the published national 12-month retention rate over the same period was 67% [29].

### Changing measurements and indicators

HIV indicators have frequently evolved to align with changing norms of prevention, diagnosis and treatment. These changing indicators have made conducting time-series analyses difficult. For example, in both the Mozambique and Cote d'Ivoire studies, the proportion of infants who received polymerase chain reaction (PCR) testing had been measured against the number of infants of HIV-positive mothers who had been registered in care. When PEPFAR changed denominators to measure infant PCR testing against all HIV-positive mothers, the proportion of infants PCR tested dropped by nearly half. Different numerator variables for HIV testing in antenatal care gave quite different results when measured against the same denominator (first antenatal visits). When the numerator included all HIV tests in antenatal care, the aggregate reported proportion of women tested exceeded 105%. When the numerator was limited to HIV tests done among women attending first or second antenatal visits, the reported proportion of women tested dropped to 89%.

The proportion of patients retained in ART has been especially difficult to assess due to constantly changing criteria for retention. Different sources of data (patient charts, pharmacy records and/or community outreach registries) change frequently – and different recording systems report different retention rates. Since 2013, PEPFAR partners have measured retention in ART by calculating the percent of adults and children known to be alive and on treatment 12 months after initiation of ART. In both Mozambique and Cote d'Ivoire, the number of people who initiated ART in the 12 months prior to the beginning of the reporting period frequently does not correspond to the reports of the same indicator in the previous year report, usually increasing the reported proportion of people retained in ART.

### Differential utilization and patterns of services

The Mozambique linkages study reported two urban districts with a high proportion of patients lost to ART follow-up, some of whom might have simply changed their care to one of the other ART facilities in the same city. Such unrecorded transfers are more likely in urban than in rural areas; thus, the divergence of reported retention from true retention may be greater in urban areas. Transfer rates were reported to be as high as 20% in one urban facility in the Mozambique study, but rare in rural facilities. When transfers are not accounted for in ART retention calculations, reported retention rates may underestimate the true retention.

The Cote d'Ivoire PMTCT study data suggested that proportions of women who were tested for HIV in private health facilities were substantially lower than in government facilities. Although some of the larger private facilities report to the national system, many of the smaller private facilities do not routinely report. It is possible that pregnant women who elect not to be HIV tested simply attend these private clinics for their first visits to “opt out.” If this is true, our reported HIV testing proportions may be substantially overestimated, especially in urban areas where there are higher numbers of private clinics.

### Linkage of data

In the Uganda and Rwanda studies, multiple registries across different HIV services or health facilities made it difficult to link individual patient entries among the registries, or to track outcomes of mother-baby pairs. Occasionally, unique patient identification numbers link data sources. In other cases, it has been difficult to link records given the number of patients with similar names. In Mozambique, because patient codes were different among those who tested HIV positive and those who registered in HIV care, no direct linkages regarding HIV patient follow-up, including transfers, could be made. The Rwanda study highlighted the limitation in tracking each mother-baby pair and mothers from ANC to labour and delivery and into postpartum care, as delivery often took place in a different facility. Key information often was not recorded in a patient chart, forcing health workers (or study teams) to search registries for critical information pertaining to that patient.

### Other considerations

Time gaps between numerators and denominators were frequently seen in Cote d'Ivoire and Mozambique. Late entries of laboratory tests received after the reporting period when the patient was counted may have underestimated the true proportion of laboratory tests performed in HIV services. Challenges archiving paper-based charts likely contributed to underestimates of patient retention in both Mozambique and Cote d'Ivoire. Storage units were rare, and filing cabinets were overflowing with charts, making it difficult to find charts during the study team visits. Routine review of either paper-based and EMR records was infrequently documented.

Table 1 below summarizes some of the challenges encountered in these analyses of secondary data. Many challenges and their causes are similar for both RHIS and PHI.

**Table 1:** Illustrative challenges related to recording and use of secondary data from routine health information systems (RHIS) and personal health information (PHI)

Challenges of recording and using RHIS	Challenges of recording and using PHI (paper charts and EMR)
Excessive registries, indicators, and time burden on health workers Inconsistent data entry by multiple people Limited accountability for quality of registering Registry of information in two or more books Unlinked information among registries Inadequate archives for registry books Infrequent data use for decision-making and analysis at facilities and districts Infrequent sharing of data to cross-check Infrequent data quality assurance checks Numerator**–**denominator incompatibility Differential patterns of utilization	Time burden on providers who fill out (or don't fill out) charts Culture of lax charting Limited accountability for quality of charting Duplicate registry of information by providers Unlinked information across registries and charts Inadequate archives for paper charts Infrequent chart review for improvement of patient care Infrequent EMR maintenance Infrequent data quality assurance checks

## Discussion

Many of the challenges that undermine the effective use of both RHIS and PHI were related to the processes and context of data collection. The many duplicative and unlinked registries, multiplicity and changing of indicators and the poor integration of data collection systems across different programmes, all contributed to weak data. Other contributors included the lack of accountability for data quality at facility levels, the substantial burden on health workers to collect and analyze the data, the weak transmission of data upward through health systems and the limited review or use of data for decision-making and policy.

These problems were remarkably consistent among all four countries – countries that represent Anglophone, Francophone and Lusophone cultures and approaches to health care. Their experiences with the burdens and challenges of data collection and analysis are likely generalizable to other countries, especially to those that report on PEPFAR indicators.

Indicators for HIV/AIDS programme tracking, monitoring and evaluation change for numerous, often important, reasons. Multiple stakeholders, with the best of intentions, periodically develop newer and better data collection registers and forms, typically resulting in more indicators added than subtracted. Donors and partners engaged in vertical programmes, operating across many countries, can be relatively blind to other programmes or to the overall RHIS and PHI needs at health facility levels. PEPFAR alone has over 500 indicators for HIV programmes, many of which are supposed to be collected monthly for every health facility. It is no surprise that large numbers of data fields are incomplete in these settings. Over-worked health workers might understandably place less value on accuracy than the recipients of the data, particularly where the use and utility of these data is unclear or unknown by the health care worker who is collecting the data. Reporting is still seen as externally imposed in most places and understanding of the value of data to service delivery is often not clear.

Our review has led to a number of key recommendations for HIV/AIDS data systems going forward. First, data sources, analysis and reporting should be simplified. This will mean fewer indicators, registries and reports – a recommendation that the WHO highlighted in their 2015 *Consolidated Strategic Information Guidelines for HIV in the Health Sector* [30]. The WHO has advocated for only 10 global indicators and a total of 50 targeted indicators at the national level. Achieving this would require stakeholders involved in different programmes to spend substantial time together coordinating indicators, with an eye on the diverse demands of health facility staff, and to work out broad-based RHIS and EMR that can function across programmes and settings. Furthermore, all stakeholders should work to minimize frequent changes to established indicators. When indicators are changed, those enacting changes should clearly describe how these changes might impact the continuity of tracking key indicators over time – or provide guidance on how to maintain optimal indicator continuity.

Second, collaborative RHIS data audits should be built into the overall system to determine the magnitude of data reporting failure, guide site-specific improvements in data management and involve those who generate facility-level data in understanding their own data. Data quality audits and participatory data-use interventions have been carried out across many countries with proven success – not only improving accuracy and completeness of RHIS and PHI, but also enhancing the value and use of data for quality improvement [31,32]. Routine data review meetings, patient chart review and use of data dashboards have been shown to be effective to increase data quality, ownership and use of data for decision-making [33,34]. Major efforts have been carried out in many countries to improve district- and facility-level data, including implanting open source software for district health information systems (DHIS2) [35–39]. However, systems such as DHIS2 have only been effectively nationalized in a few countries [40]. More often, DHIS2 has been implemented at sub-national levels with the support of donors and NGOs, without strong support from the central Ministry level [41]. It is unclear if innovative approaches that are primarily implemented by external entities will solve the fundamental issues we identified, especially the excessive indicators, ability to link data, lack of involvement of health staff in data use and quality assurance data generated at the facility level. Without central Ministry of Health coordination, the issues we highlighted above around linkages of data may only be exacerbated by a patchwork of data systems and indicators across clinics or districts. All stakeholders – national policymakers, facility staff, district and provincial managers, funders and implementing partners – should ensure substantial HIV-related funds are dedicated to ensuring that these data-related activities are seen as an essential element of the overall Primary Health Care system.

Last, RHIS and PHI generated by government, donors and partners should be widely accessible, and where possible, open source, to ensure that the data are consistent, reviewed and appropriately analyzed by all stakeholders to drive data-driven decision-making. As these data are often the only data sources which are actionable at the district and health facility levels, increased investments in these systems are necessary for quality improvement. The current situation, whereby RHIS and PHI are not openly available for secondary analyses, limits their use and continues the status quo whereby most analyses use infrequent community surveys, which fail to provide health facility or district data where action is needed. It has been demonstrated that RHIS data can be paired with community surveys or other HIV-related data sources to estimate who is currently being missed by targeted facility-based ART delivery [30]. Innovative efforts to improve health facility catchment area estimation can also help target investments to areas with high HIV burden and low ART coverage [31]. With increased access, such methods to pair RHIS and PHI data with community sample surveys and/or census data could also help understand which areas of the health system are failing to reach a representative sample of those testing HIV positive.

## Conclusions

RHIS and PHI data provide substantial opportunities for investigators, health workers and policymakers to understand health service coverage and use data for real-time health system decision-making. However, the poor and/or variable data quality of RHIS and PHI, along with frequent changes in indicators, difficulties in linking individual-level data over time and data sources, and differential service utilization, all present considerable challenges for analysis and use of these data to improve HIV programmes. Based on our review, we recommend concerted efforts by all stakeholders to simplify indicators, routine reporting and data collection efforts along with focused efforts to maintain key indicators unchanged over time to allow easy monitoring of programme success or failure. These simplified data sources should undergo routine data quality audits and chart reviews, paired with the explicit engagement of health workers and managers in the use of data for analysis and decision-making. With these investments, and the continued expansion of data availability through the open-access movement, RHIS and PHI data will be more widely available and useful for high-quality monitoring and evaluation of HIV-related programmes at the health facility, district, national, and international policymaker levels.
